# Survival, stigma and structural barriers: a qualitative study exploring the oral health experiences of women attending sex work support services in Liverpool

**DOI:** 10.1038/s41415-026-0079-8

**Published:** 2026-10-09

**Authors:** Janine Doughty, Gemma Ahearne, Posey Jolly, Stef Ryder-Walker, Heni Nagy, Amy Rawsthorne, Isolde Godfrey, Jude McMaster, Julie Smedley

**Affiliations:** 081179685182960168529 School of Dentistry, University of Liverpool, Liverpool, UK; Liverpool University Dental Hospital, Liverpool, United Kingdom; 565772358266277321784 School of Law and Social Justice, University of Liverpool, Liverpool, United Kingdom; 218563491581377705778 Woven Ink, Oxford, United Kingdom; 493160329816309195335 Pearl´s Project, Liverpool, United Kingdom; 625757821350120477751 Change Grow Live, Liverpool, United Kingdom

## Abstract

**Aims** Globally, sex workers experience significant oral health inequalities, including higher levels of dental decay, tooth loss, and trauma. Sex workers face intersecting challenges including homelessness, addiction, and stigma. In the UK, no qualitative research has explored the oral health experiences of sex workers. The aim of this research was to understand the oral health experiences and needs of women attending sex work services in Liverpool, to identify perceived barriers to care, and to explore acceptable approaches to improving services through participatory engagement.

**Methods** A qualitative participatory study was conducted with adults with lived experience of using sex worker support services in Liverpool. Twenty-one semi-structured interviews were undertaken following opportunistic and snowball sampling through community support services. Interviews were analysed using a framework approach to generate inductive codes and themes. The themes were translated into professional illustrations which supported women to engage in the data co-interpretation process.

**Results** Participants described lives shaped by violence, homelessness, and poly-substance use, with oral health positioned low within daily survival priorities. Drug use, high-sugar diets, xerostomia, vomiting, and disrupted self-care contributed to rapid dental deterioration. Poor oral health produced significant physical pain, impaired function, and profound shame linked to intersecting stigmas of sex work, addiction, and visible dental damage. Access to comprehensive National Health Service dental care was widely perceived as impossible; most care was urgent or self-managed, including DIY (do it yourself) dentistry. Despite this, women expressed strong motivation for restorative care and valued non-judgemental services.

**Conclusions** Women who attended sex worker-specific support services in Liverpool experienced severe, intersecting oral health harms compounded by structural barriers to comprehensive rehabilitative oral care. Trauma-informed, flexible, and comprehensive dental services co-designed with women are required to reduce inequalities and stigma.

**Supplementary Information:**

Zusatzmaterial online: Zu diesem Beitrag sind unter https://doi.org/10.1038/s41415-026-0079-8 für autorisierte Leser zusätzliche Dateien abrufbar.

## Key points

- Poor oral health driven by intersecting factors including xerostomia, high-sugar diets, vomiting, and disrupted self-care functions as a visible marker of marginalisation, reinforcing stigma related to sex work, addiction, and poverty.
- Oral health impacts extend beyond pain and function to include shame, reduced self-worth, and impaired social and intimate relationships.
- Structural barriers such as service inflexibility, geographic distance, and appointment systems do not align with the realities of people experiencing complex vulnerabilities.

**Supplementary Information:**

Zusatzmaterial online: Zu diesem Beitrag sind unter https://doi.org/10.1038/s41415-026-0079-8 für autorisierte Leser zusätzliche Dateien abrufbar.

## Introduction

Sex work can be understood as a broad spectrum of commercial sexual exchange, this can be financial, favour, or shelter. There are around 102,000 (confidence range 75,000–130,000) sex workers in the UK, some working on-street (the smallest part of the UK sex trade), others working in sexual entertainment venues and indoor premises such as brothels and saunas, and fluidity between the markets.^1^ Much sex work has moved online and now accounts for 60,000–80,000 sex workers.^1^ Street-based sex workers can be understood as survival workers; many are living with addiction and complex needs. Survival-based sex work is the exchange of sexual acts for immediate essential needs like food, shelter or substances and is often driven by extreme poverty or homelessness.^2^ In addition to this, we also engage with Bowen's work on the duality of sex work, and the transient and fluid nature of both working in the sex industry at all, and of moving between different markets within the sex industry.^3^ This article describes novel research undertaken by a multidisciplinary team across dental practitioners and researchers and criminology. The paper does not take a moral stance on the exchange of sexual services for money, but rather, takes a pragmatic approach to harm reduction. *For awareness, this article contains discussions of sexual violence and drug/alcohol use.*

## Sex worker oral health

Internationally, sex workers experience significant disparities in oral and general health when compared with the general public; they experience more dental decay, have more missing teeth, poorer oral hygiene, higher incidence of temporomandibular joint dysfunction, more frequently engage in smoking and alcohol use increasing oral cancer risk and are at higher risk of sexually transmitted oral infections.^4^^,^^5^^,^^6^^,^^7^ Further, sex workers are disproportionately at risk of facial injury and dental trauma. A recent review on inequalities in oral health found no papers published on the topic of sex workers in England, demonstrating the urgent need for this gap in research to be remedied.^8^ Sex workers are a highly stigmatised population, which creates numerous barriers to engaging with support. Doughty *et al.* (2023) describe how stigmatised characteristics intertwine with visibly poor oral health to amplify oral health stigma and discrimination.^9^ Sex workers specifically experience body dissatisfaction and low self-esteem related to dental damage.^10^ Despite the wealth of international evidence to suggest that sex workers have considerably poorer oral health than the general population, there has been no research in the UK to understand the oral health experiences and needs of sex workers and ways to address them. The aim of this study was to understand the oral health experiences and needs of women attending sex work services in Liverpool, to identify perceived barriers to care, and to explore acceptable approaches to improving services through participatory engagement. This participatory research process will provide insight and direction for future studies and frameworks around sex worker oral health.

## Methods

The consolidated criteria for reporting qualitative research (COREQ) have been used to comprehensively report the methods used during this study.^11^

This study was granted ethical approval by the University of Liverpool Ethics Committee (reference: 15705).

### Qualitative data collection

Participatory qualitative methods included a multi-staged method as follows:

- In-depth interviews undertaken with participants who either self-identified as being a sex worker and/or were engaging with sex worker charity services. Participants were recruited from two sites in Liverpool both in the day and at night
- A preliminary coding process was undertaken
- Early codes and data saturation were assessed with a further eight participants who undertook in-depth interviews
- Thereafter, a coding matrix was developed and used as a conversational prompt with a further five participants during interviews, women prioritised each of the codes by their relevance and/or the severity of impact
- Pencil-drawing illustrations were designed by a professional illustrator (Supplementary Material 1). These pencils drawings were co-created with thirteen women and were also used as member-checking tool to establish the appropriateness of our interpretation. Their perspectives were captured using post-it notes, field notes and using hearts to vote on the colour scheme. These anonymous shorthand notes were then typed up and shared with the illustrator to develop the final iteration of the illustrations
- The illustrations and findings from the study formed part of an exhibition event sharing outcomes with local organisations.

Interviews were conducted and an illustration member-checking session were undertaken between August and November 2025. Access to sites was permitted by gatekeepers to the services who supported our outreach attendance. All participants were provided with a £25 Amazon gift voucher, oral health pack, and lunch for each session attended. The interviews used a semi-structured approach facilitated by open-ended questions. The topic guide was developed by JD and GA and reviewed by the sex worker support service and gatekeepers working with and advocating for sex workers (JS, JM). JD, GA, PJ, SRW and AR were all involved in interviewing, taking field notes, analysing and interpreting the data, member checking and participatory development of the illustrations. Data were triangulated by collecting data from different sites at different times of day, involving multiple data coders, taking field notes of observations from the interviews, and using multiple data collection methods including the member-checking of illustrations.

### Sample size

In total, 21 participants were interviewed; participants were living in temporary hostel accommodation and were accessing sex worker-specific services. Interviews were ceased once sufficient richness of data had been achieved to answer the research question/achieve the aims of the participatory study. 

### Sampling strategy

Snowballing and opportunistic sampling were used together to identify and select participants who self-identified as having experience of sex work or used sex worker support services. Snowballing sampling utilised the networks and connections of gatekeepers to link to other sex worker charities and services. We also recruited through women who we built relationships with, specifically one key former sex worker who now volunteered with the service. She informed other women of the study and explained how she had experienced taking part. Due to the drop-in nature of the services and the potential for transience within the sex worker population, opportunistic sampling was also employed. We did not sample for other characteristics beyond participants being over the age of 18 and having lived experience of sex work or use of support services. Some women were not willing to disclose sex working verbally or preferred to be described as shoplifter rather than sex worker for example; women using sex-worker services who did not disclose sex working were still included in the study if they understood the purpose of the study and were willing to participate. No women were coerced into explicitly disclosing sex working to participate in the study.

### Data collection and analysis

Interviews were conducted by JD, GA, AR, PJ and SRW. Data were recorded on a password-protected device and the anonymised transcripts were saved on the University of Liverpool password-protected computer. Analysis of the qualitative data used a seven-step framework approach to undertake inductive coding of the transcripts: transcription, familiarisation, independent coding (JD, AR, SRW, PJ, HN), developing framework, applying framework, charting data into framework matrix and interpreting data.^12^ Initial codes were generated; these were member-checked with the women using the services. Similar codes were grouped together to create the initial framework which was applied across all transcripts (JD, AR, SRW, PJ, HN) before summarising transcripts into the framework (HN and JD). Disagreements were resolved through a discussion process. An audit trail was maintained throughout the coding process which included early iterations of the framework, minutes of discussions and copies of member-checking of the codes and illustrations reflective of the emerging themes. Microsoft Word and Excel software were used to manage the data and the analysis processes. The data were presented as themes and sub-themes. Women were presented with transcripts and written summaries of the findings but did not engage with these documents. Therefore, co-developed illustrations were used as a method of co-analysis with the women to challenge traditional knowledge hierarchies and overcome challenges of low levels of literacy and short attention windows that precluded co-analyse in traditional text format.

### Theoretical framework

The frameworks underpinning this participatory methods study are twofold: feminist theory which emphasises intersectionality, reflexivity, and addressing systemic inequalities, particularly those affecting marginalised identities. Feminist theory recognises and challenges gendered and intersecting forms of stigma and oppression including the stigmas of oral health and sex work. Community-Based Participatory Research Framework emphasises equal partnerships between researchers and participants, shared decision-making, and practical outcomes.^13^ There is a rich and valuable history of using participatory action research with sex working communities.^14^

### Eligibility criteria

The following eligibility criteria were used:

- Adults aged 18 years and over
- Adults with lived experience of sex work or using sex-worker support services in Liverpool
- Sufficient understanding of English to participate in the interviews
- Sufficient capacity to consent to participation.

### Consent

Written consent was obtained before the participant underwent any activities that were specifically for the purposes of the study. Due to risk behaviours such as drug and alcohol use being common among on-street sex workers, a clear definition of capacity to consent was agreed as follows. A capable person will: understand the purpose and nature of the research, understand what the research involves, its benefits (or lack of benefits), risks and burdens, understand the alternatives to taking part, be able to retain the information long enough to make an effective decision, be able to make a free choice, be capable of making this decision at the time it needs to be made (though their capacity may fluctuate, and they may be capable of making some decisions but not others depending on their complexity). Where participants were capable of consenting for themselves but were particularly susceptible to coercion, participants were informed how their data and anonymity were protected. Capacity was assessed before the consent process by both the service gatekeepers and researchers. The research team engaged in informal conversation before the interviews, sought support and advice from the service gatekeeper about any known medical or social issues that might impair consent, engaged in dialogue around the study processes and confirmed understanding verbally as well as by signing. Signs of slurring, drowsiness, confusion, inability to retain information short-term, distress or progressive reduction in response before or during interviews was carefully monitored throughout and responded to (distress protocol: online Supplementary Material 2). Interviews were undertaken within the usual operating hours of the services; participants who did not have capacity to consent or became distressed during interviews were referred to the site gatekeeper/lead who would then decide the appropriate course of action based on their knowledge of the individual women and their circumstances. We provided an information leaflet that contained signposting for local mental health services. All potential participants were provided with written information sheets, signposting, study contact details, and a recording was available to overcome literacy issues. All data were anonymised. Pseudonyms have been used to humanise the selected quotes from the study, avoiding any matching initials to the participants.

### Research team and reflexivity

JD and GA are postdoctoral researchers in dentistry and criminology, respectively. Other members of the research team included a dental research nurse, dental students, and sex worker support service leads. All researchers were white and female. Three researchers originated from Liverpool. JS and JM knew the participants well as they had been delivering the sex worker support services for several years, the remainder of the team did not know the women before the interviews. Participants were informed of all researchers' professional roles and the purpose of the study. We discussed implicit biases during the analysis process including relative privilege by virtue of education and background. We undertook multiple member co-checking processes to ensure that our interpretation was consistent with the perspectives of the women.

## Results

There were 21 participants who took part in in-depth interviews and were included in the final analysis (Table 1). Eight participants were recruited in the first round, a further eight in the second round of interviews, and five in the third round. One participant was ineligible to participate due to impaired capacity to consent due to mental ill health; no data from this person were included in the study. Interpretation of the data generated five themes, each described in detail below.

**Table 1:** Participant demographics (total participants n = 21)

**Characteristic**	**n**	**%**
**Age group (years)**		
25–34	1	4.8
35–44	12	57.1
45–54	8	38.1
**Gender**		
Women	20	95.2
Women (transgender)	1	4.8
**Ethnicity**		
White British	18	85.7
Black (A or C)	1	4.8
Mixed (multi-ethnic)	1	4.8
White Roma/Traveller	1	4.8
Sex work type*		
Outdoor	13	61.9
Indoor	8	38.1
Escorting	7	33.3
Online/Remote	5	23.8
Transactional	2	9.5
Dancing/SEV	1	4.8
Not willing to disclose	3	14.3%
**Time in sex work**		
<6 months	4	19.0
6–12 months	3	14.3
1–3 years	1	4.8
3–5 years	0	0.0
5–10 years	4	19.0
>10 years	6	28.6
Not willing to disclose	3	14.3
SEV, Sexual Entertainment Venues. *Participants could select multiple sex work categories. Denominator for each category n = 21.		

### Sex workers and survival: a constant threat of violence, instability and addiction

The women provided deep insights around the complexities of their daily lives, their vulnerabilities, and the threats posed by the environments in which they lived and worked. This information demonstrated how oral health concerns ranked as a low priority within their lived reality. All subsequent themes should be interpreted with this theme as a contextual backdrop.

Many sex workers described historical and present threats in the environments in which they survived. All the women were living in temporary accommodation in hostels local to the services. Most women were using at least one drug, often women used multiple drugs (i.e., heroin, crack, spice); many also smoked and used alcohol. Some women spoke indifferently of sexual violence, demonstrating the frequency and normalisation of violence in the women's daily lives. For example, women spoke of clients removing condoms and performing unprotected ejaculation without consent during oral and vaginal sex and some women spoke of having been raped or suffered domestic violence. The women told us how they had not reported these crimes to police for fear of not being believed or retribution from clients. Two women told of a horrific act of violence against a sex worker, where they described them ‘*being murdered,* *and they are getting chopped up in wheelie bins and stuff*' [Emma]. Ahearne coins this an ‘almost violence', a looming, haunting threat of what might happen to sex workers.^15^ The historical cases of the murders of Hanane Parry, Pauline Stephen and Chantel Taylor are passed through generations of sex-working women.^16^^,^^17^

Women spoke of children and grandchildren with whom they had little or no contact or who were themselves drug users. The women commonly used formal language to describe other sex workers (‘associates') thereby distancing themselves from both other sex workers as well as the stigma associated with sex-working:

*‘I don't speak to my family. I haven't got no friends. They're just associates, from there and that's it.…When I was having my birthday last week. I never took a picture. I didn't even have a photo with anyone. That's bad. I just don't like the way I loo*k. *It's heavy'* [Leanne].

Acute mental health illnesses were disclosed by participants, including suicide attempts. During the interviews, at least three women cried as they reflected on their present life circumstances and how they experienced their oral health. One woman described the hostel as ‘*disgusting. It's full of cockroaches. Oh, it's disgusting. It's wild*' [Claire]. Another, who was new to sex work, reported that being around other sex workers in the hostel was the reason she had become involved in sex work. One participant described her journey of detoxing from drugs; being relocated to a hostel around other drug users led her to start using again. She described hostels as: ‘*the worst place possible [for a recovering addict]'* [Leanne]. Many women reported experiences of homelessness and losing friends to perishing on the streets:

*‘I've been homeless, I've been on streets as well, yeah. Hmm. I was for a few years, but as I'm getting older, I know that I wouldn't survive it no more. You know, if I was to go on the street again, I wouldn't survive'* [Emma].

### A downward spiral of oral health harms and experiences

Several women described the place of oral health in their childhood. While some women had positive and supportive experiences of childhood and were encouraged to attend the dentist by their parents, others were raised in homes where parents used drugs and oral health was neglected:

*‘There was just no focus on me brushing my teeth at all [in childhood], because my mum was on drugs too, so we was lucky to have a loaf of bread in'* [Sophie].

Some women were using drugs and alcohol by their early teenage years. Some women described how the loss of a parent who provided organisational support to attend dental appointments led to a downward spiral in oral health, exacerbating drug use:

*‘My dad died when he was 32 from injecting, and his teeth, he was 32, and I remember his teeth, I was only nine, but I remember going to the pictures and him getting a packet of yellow M&M's, and he had to eat them at the front, and he was only 32'* [Sophie].

The most common causes of poor oral health were linked to drug use, high sugar diets, domestic violence and vomiting. Women explained that heroin led to sugar cravings and crack caused an extremely dry mouth and loss of appetite. Many women knew that methadone with sugar could impact oral health, and that methadone caused a dry mouth. Chaotic eating patterns and eating disorders were common. For example, one woman described adding a jar of Nutella to milk as her main source of food:

*‘I just eat when I can and when it's available, to be honest. I have gone like a couple of, maybe, three days without anything when I've been on my low points. And then there's days where it's been like buffets and there's all you can eat. It's just, it varies day to day. Yeah. The life that you live around here. It's crazy.*' [Amy].

Dental health deteriorated rapidly. Some women described their teeth crumbling away within a few years of starting to use drugs. Brushing teeth was commonly forgotten or didn't feel important when using drugs. Several women who were missing multiple teeth felt that there was no point in brushing their teeth when they were already beyond repair. Very few women mentioned money as an issue relative to toothbrushes and paste; some explained that the hostel provided toothbrush and paste. However, one woman with experience of homelessness described the difficulties of brushing teeth when homeless because of having to carry all of one's belongings and the inconveniences of carrying a wet toothbrush. Women who wore dentures commonly reported that they were lost or stolen in the hostel. Oral health problems harmed women physically, mentally and socially. Commonly experienced harms included dental abscesses, pain, missing many or all teeth and crumbling teeth. One woman described how her teeth would spontaneously fall out into her hand. Broken and sharp teeth had caused issues with oral sex. Multiple women described oral health as affecting sex work. Some clients complained that they felt as though they were being bitten during oral sex because of sharp teeth:

*‘Sharp bits, all my tongue underneath, if you lift my tongue up, even now, it's stinging and, got a big blister under it because it's sharp, sharp. I've had to nail file it'* [Sophie].

### Dirt: shame and stereotypes at the intersection of sex work and oral health

The combination of physical and psychoanalytical interpretations of ‘dirt' was embedded throughout the conversations with women – sex workers' bodies disposed of as dirt in wheelie bins, the dirt of hostel environments in which the women existed, and their crumbling and decayed teeth visible as an inescapable marker of the ‘dirt' of drug use and sex work. Ahearne argues that sex workers are socially constructed as ‘dirty' or ‘dangerous' which leads to the state abandoning the standard protections usually afforded to citizens.^15^ Oral health has been described as provoking disgust, a powerful contagion avoidance emotion.^9^ Many women understood their teeth as unattractive to onlookers; they felt gender amplified this. Some felt ashamed or embarrassed and had low confidence because of their teeth. Many women did not want to look in the mirror, covered their teeth with their hair or their hands and avoided kissing their partners when they felt self-conscious about their oral health:

*‘It's horrible. It's embarrassing. I don't like talking or nothing or even eating. Because everyone, that's the first thing people look at'* [Dannielle]

*‘I feel ashamed showing them my teeth. I'm embarrassed. Yeah, I wear a scarf. Yeah, I never smile. I try not to anyway. Oh, it's a very bad impact on me. Yeah. It's embarrassing, isn't it?'* [Michelle].

Women commonly shared that poor dental appearance elicited stereotypes and labelling and described how even if they detoxed from drugs, they would still look like a drug user because of the appearance of their teeth:

‘Yeah, it's the biggest giveaway in the world that you're a smack head, you know. And then everyone I walk past, they just think, they're thinking, oh, f**king smack head' [Gemma].

The topic of self-worth and self-esteem was vitally important across the conversations, particularly how oral health reflected visible information about women to themselves and to onlookers. Most did not like to look in the mirror. Losing teeth was associated with a loss of sense of their self before drugs and sex-working began. Many women described themselves as feeling worthless and expressed not liking themselves:

*‘They have to clean up one day and go like “Oh, I like that part of myself or something”, it's something you can't give, I don't think, I think it's in everyone, I think you just have to find it. Encourage it, plant it, like a seed, plant it, say things, notice things, if someone's got a nice thing say “oh that's a nice thing”. It's hard making people look at themselves'* [Laura].

### Dental service availability and design are commonly mismatched to dental access and oral health needs

Almost all women described the impossibility of gaining access to a dentist for comprehensive dental care to restore their oral health and appearance. Women described accessing urgent dental services to address issues of pain and where they could not find a dentist, some self-medicated with drugs to manage the pain:

*‘I can't get a dentist, I can't get a dentist for love nor money, I just can't get a dentist'* [Leanne].

Several women described do it yourself (DIY) dentistry to file down sharp teeth. Some women used the dental bus provided by the charity Dentaid:

*‘I like the van, I basically like the [dental] van. By the way, it was nice, it was very nice on the van. It was very friendly, very nice, very clean and cleanliness in there. Just nice people. Nice environment, nice people. Just giving me good advice'* [Natalie].

However, only urgent dental treatments were available, which addressed acute issues but did not address the women's comprehensive dental care needs:

*‘I'm supposed to be getting a little bit of money in the next couple of weeks, and I want to go and get my teeth done, the first thing I want to do is to try and get my teeth done. I just don't even know where to start'* [Sophie].

Access to a dentist felt like an impossibility:

‘Phone, phone, phone, “can I register, can I register”, everywhere. I think I've phoned every f**king dentist in Liverpool. Even lied about where I live. I've even lied that I live around the corner. I've found postcodes, whatever, I've tried, can never get a f**king dentist, I can't get a dentist' [Laura].

Even when women gained access to a dentist, they described long waiting lists, financial issues, timing challenges, and travel distances that posed barriers to getting dental treatment. Missing appointments and losing access to a dentist was reported by a few women, despite legitimate reasons for missing the appointment such as hospitalisation:

‘It [the dental service] was miles away. It was in five miles away [from the location]. It was miles away and me nan had to drive. And she was wanting me to go at f**king 10 o'clock in the morning' [Jade].

A few women felt prejudiced by dentists. Others described being treated kindly by dentists and explained that the gender of a dentist was not important to them; it was more important that the dentist was qualified and kind. Many of the women described dental phobia as a deterrent from attending the dentist:

‘Sometimes I felt that one of the dentists looked down on me and it made me feel really s**t' [Emma].

Several women had previously accessed prison dental services, which they described as helpful; however, comprehensive dental care was only available to those with longer sentences. Women with shorter sentences reported being seen for emergency dental care only. Women strongly wanted complete care, including the provision of dentures where relevant, and wanted help to find a dentist; those who had received a course of treatment from a dentist were very positive about their experiences and valued the care they received. One woman who had stopped using drugs and sex working proudly showed her dentures and explained how she could now smile. During the interviews, several women asked for support to find a dentist from the research team and the charity support workers. Though not a priority for all women, some women wanted better explanations from dental professionals and more understanding when appointments were missed.

### Supporting and motivating women accessing sex worker services toward improving oral health

Though most women who were interviewed suffered severe oral health harms, there were three women who had good oral hygiene and had suffered from very few dental issues. Reasons that the women gave to explain their good oral health included having had good oral hygiene reinforced as a child, prioritising daily oral health, use of only one drug rather than a combination of drugs, fastidious use of condoms during oral sex, and having access to a dentist and attending regularly:

*‘We had to go religiously, me mum took us religiously when we were kids, you know, and yeah there used to be good experiences there [at the dentist]'* [Emma]

*‘I think it's [having healthy teeth] just because I'm using the one drug [crack]. If I was using heroin and that, it would probably be a different story'* [Amy].

Women who were anxious about the dentist described how having a friend or support worker attend with them would support them to have the dental treatment they needed. For some women the motivation to restore their oral health was closely tied to stopping drug use and sex work and a desire for their families to see their dental health looking good:

*‘A million percent [want to get teeth sorted] because if not I'm not going to go out and live my life. Yeah. With my teeth like this. It's not going to happen. I know I won't. I don't want to see my daughter like this'* [Gemma].

## Discussion

Taken together, the findings from this study suggest that oral health among women who attend sex worker support services and who are involved in on-street sex work, cannot be understood solely through individual behaviours, but must be interpreted through a lens of survival, stigma and structural barriers. In this study, oral health cemented stigma through visible and irreversible impacts of poverty, drug use and self-neglect on the women's teeth. Oral health functioned as a marker of inequality. The participants in this study embodied multiple intersecting stigmas: sex work stigma,^18^ drug and alcohol addiction stigma^19^ and oral health stigma.^9^ Missing or visibly decayed teeth lead to inferences of poverty, self-neglect and drug use.^20^

Most women in this study were using multiple drugs and alcohol. Drug use has consistently been reported as harmful for oral health through multiple mechanisms including xerostomia (dry mouth), increased intake of sugary foods and drinks, jaw clenching and poorer oral hygiene linked to dental decay, tooth wear, periodontal disease, tooth loss and general dental neglect.^21^^,^^22^^,^^23^ Opioid use specifically was associated with worse oral health ratings over time.^24^ Drug use stigma can result in labelling, social exclusion, stereotyping and discrimination that legitimise hostility, substandard care, blaming individuals and deeming them unworthy.^25^

Coles and Freeman describe homeless people's journey of oral health from deconstruction of self-care, to the construction and maintenance of the dentition and ultimately reclamation of oral health and life.^26^ The women in this study were at various stages in the deconstruction of their dentition. One woman who had left sex work and stopped drug use had sought treatment for dentures which had improved her sense of self and the life that she was embarking on enabling her to now volunteer as a support for other women using the service. Many women hid their mouths with their hair, scarves, their hands, did not smile or refused to reconnect with their family because of fear of being judged about their oral health. Dental shame has been suggested as a critical lens for understanding and addressing the complex interplay between personal, social, economic, cultural and systemic factors contributing to oral health challenges.^27^

In this study, few women mentioned being treated differently or with judgement from dental professionals and most actively desired access to a dentist. However, in wider literature, fear of judgement from healthcare providers has been reported as a major factor responsible for unmet health needs.^28^ People who are homeless, have addictions or mental ill health are reported to feel powerless when interacting with health and care systems.^25^

Women attending sex worker support services reported issues with accessing a National Health Service (NHS) dentist as a strong and repeated concern. In recent years in England, more than 97% of people who did not have access to an NHS dentist failed to obtain access when they attempted to.^29^ A priority for the women in this study was the desire for comprehensive dental care that enabled them to be free from the stereotypes provoked by visible dental destruction. Urgent issues were management by self-medicating, DIY dentistry or by seeking care from an urgent dental service provider. Many wanted access to oral rehabilitation to address the appearance that had occurred as a result of drug use.

When women were able to access a dentist, they faced multiple barriers including transportation issues, competing hospital appointments, incarceration and timing issues with early morning appointments. These same factors have consistently been highlighted as a mismatch between the operations of mainstream dental services and the needs of inclusion oral health groups that makes it ‘difficult to support patients with a ‘chaotic lifestyle and changing living circumstances'*.*^30^ Globally, interventions that have been used to improve the health of sex workers include education and empowerment, drug harm reduction services, sexual and reproductive care services, and multi-component clinics and outreach activity.^31^ In England, drop-in models provided by charities and community dental services which offer flexible appointment times and mobile dental units have been highlighted as models of care that facilitate access.^30^^,^^32^

### Strength and limitations of the study

This study presents the first qualitative exploration of the oral health of women involved in sex work or using sex worker support services in the UK. The qualitative methodology provided in-depth insight into the oral health experiences of a population that remains underrepresented in oral health research. Collaboration with trusted community organisations supporting women involved in sex work or using sex worker services also facilitated engagement with participants who might otherwise be excluded from research. The use of co-interpretation of the study findings using participatory and arts-based methods is novel and a useful approach for populations with literacy issues. However, several limitations should be considered when interpreting the findings. Most participants were white British women living and working in Liverpool. The findings therefore do not reflect the experiences of women from diverse ethnic groups, trafficked sex workers or the views of male sex workers. Most women in this study used drugs and were involved in face-to-face, as opposed to online sex working. Though both face-to-face and online sex work happens for financial reasons, on-street sex work is more likely to be associated with pressure to prostitute for drugs which has implications for oral health. Oral health conditions were self-reported rather than clinically assessed, and accounts may therefore reflect perceptions of oral health as well as objective disease. Despite these limitations, the study offers valuable insight into how oral health intersects with stigma, addiction and structural barriers, and highlights important areas for service innovation and future research.

### Recommendations for practice, policy and research

The findings of this study highlight NHS dental system failure, specifically the structural exclusion of marginalised women, highlighting a mismatch between service design and lived reality. Improving oral health outcomes for this population requires service models that move beyond traditional delivery structures. Flexible approaches such as outreach services, drop-in clinics and mobile dental units, particularly when delivered in partnership with trusted community organisations, may help reduce barriers to engagement. Practical measures within existing services may also support attendance and continuity of care, for example reminder calls arranged through support workers, hostels or outreach teams. Given the overlap between oral health needs, substance use, housing instability and mental health challenges, dental care may be most effective when delivered alongside broader health and social support. Integrating oral health into existing harm reduction, sexual health and community outreach programmes may provide a more accessible and trusted pathway to care.

Clinically, trauma-informed and stigma-aware approaches to dentistry are important to ensure that women who sell sex are not re-triggered or discriminated against when accessing dental services. Involving dental students in outreach oral health promotion could also provide opportunities to strengthen communication with inclusion health groups while delivering practical preventive advice. Within dentistry, oral health promotion is rarely framed through a harm reduction lens. Developing and communicating harm reduction approaches relevant to oral health and substance use may therefore represent an important area for future service development. Previous research has also highlighted gaps in dental professionals' communication with inclusion health groups.

Further research is needed to strengthen the evidence base in this area. Quantitative studies assessing the oral health status and treatment needs of women who sex work or use sex worker support services would complement qualitative insights and support service planning and commissioning. National research involving a broader range of sex workers, including those working online, would also provide a more comprehensive understanding of oral health needs. In addition, evaluation of innovative dental service models would help identify effective approaches to improving access and reducing oral health inequalities.

## Conclusions

This study highlights that oral health among women who sex work or use sex worker support services is shaped not only by individual behaviours but by the intersection of stigma, addiction, poverty and structural exclusion from healthcare, illustrating how deteriorating oral health can both reflect and reinforce marginalisation. Oral health influenced women's sense of self-worth, social relationships and opportunities for the future causing shame and social withdrawal. Visible dental damage symbolically signalled drug use and garnered moral judgement. Oral health barriers existed at the interface between the daily struggle for survival and structural barriers to re-engaging with dental care, highlighting misalignment between the design of mainstream dental services and the realities of people living with complex and intersecting vulnerabilities. Ultimately, addressing oral health inequalities among women who sell sex requires recognising oral health as part of broader systems of social marginalisation and recovery. Dental services that are accessible, non-judgemental and responsive to complex life circumstances have the potential not only to restore dentition but also to support a reclaiming of dignity and a pathway out of marginalisation.

## Supplementary Information

Supplementary Material 2: Distress protocol (DOCX 14KB)

Supplementary Material 1: artist illustrations of sex workers' oral health (DOCX 2MB)

## Data Availability

Anonymised data can be accessed upon written request to the corresponding author.

## References

[1] Jensen B, Weitzer R, Bennett N *et al*. Estimating the total number of sex workers in the United Kingdom: a comprehensive analysis and 2025 projection. *Sci Preprints* 2025; DOI: 10.14293/PR2199.002013.v1.

[2] Raven Bowen. Survival sex work. *Radic Hist Rev* 2024; **149:** 31–34.

[3] Bowen R. *Work, Money and Duality*. Bristol: Policy Press, 2021.

[4] Oguen-Alon T, Bilder L, Giladi H Z, Gutmacher Z, Mayer Y. Analyzing oral health conditions in sex workers – a comparative retrospective clinical and radiographic study. *Dent J* 2024; DOI: 10.3390/dj12040110. 10.3390/dj12040110 PMC11048831 38668022

[5] Mehta V, Tripathy S, Kamma P K G, Noor T, Mathur A. Oral health status of sex workers across the globe: a systematic review. *Spec Care Dent* 2025; **45:** e70068. 10.1111/scd.70068 40563216

[6] Schneider A A, Meira C G D, Galli F L, Mello A L S F, Pilati S F M. Oral health and health care in female sex workers: concomitant quantitative and qualitative approaches. *Women Health* 2021; **61:** 880–888. 10.1080/03630242.2021.1981518 34544321

[7] Eli I *et al*. Temporomandibular disorders and bruxism among sex workers – a cross sectional study. *J Clin Med* 2022; DOI: 10.3390/jcm11226622. 10.3390/jcm11226622 PMC9694590 36431098

[8] Public Health England. Inequalities in Oral Health in England. 2021. Available at https://assets.publishing.service.gov.uk/media/6051f994d3bf7f0453f7b9a9/Inequalities_in_oral_health_in_England.pdf (accessed 1 September 2026).

[9] Doughty J, Macdonald M E, Muirhead V, Freeman R. Oral health-related stigma: describing and defining a ubiquitous phenomenon. *Community Dent Oral Epidemiol* 2023; **51:** 1078–1083. 10.1111/cdoe.12893 37462247

[10] Elliott N. Meeting female street sex workers' physical and mental healthcare needs. 2019. https://www.nursingtimes.net/mental-health/meeting-female-street-sex-workers-physical-and-mental-healthcare-needs-09-12-2019/ (accessed 1 September 2026).

[11] Tong A, Sainsbury P, Craig J. Consolidated criteria for reporting qualitative research (COREQ): a 32-item checklist for interviews and focus groups. *Int J Qual Health Care* 2007; **19:** 349–357. 10.1093/intqhc/mzm042 17872937

[12] Gale N K, Heath G, Cameron E, Rashid S, Redwood S. Using the framework method for the analysis of qualitative data in multi-disciplinary health research. *BMC Med Res Methodol* 2013; **13:** 117. 10.1186/1471-2288-13-117 PMC3848812 24047204

[13] Jobe A, Stockdale K, O'Neill M. Stigma and service provision for women selling sex findings from community-based participatory research. *Ethics Soc Welf* 2022; **16:** 112–128.

[14] O'Neill, M.; Jobe, A.; Bilton, C.; Stockdale, K. Peer talk: hidden stories. a participatory research project with women who sell or swap sex in Teesside. 2017. https://durham-repository.worktribe.com/output/1635918/peer-talk-hidden-stories-a-participatory-research-project-with-women-who-sell-or-swap-sex-in-teesside (accessed 1 September 2026).

[15] Ahearne G L. ‘They only care when there's a murder on': contested perceptions of vulnerability from sex workers in prison. *Br J Community Justice* 2019; **15:** 67–76.

[16] ITV News. Mother of Woman Murdered 21 Years Ago Says She Will Fight to Keep Daughter's Killer in Prison. 2025. Available at https://www.itv.com/news/granada/2025-07-14/he-dismembered-my-daughters-body-mums-fight-to-keep-killer-imprisoned (accessed 1 September 2026).

[17] Bona E. The Killing and Mutilation of Two Vulnerable Women That Left a Community Reeling. 2018. Available at https://www.liverpoolecho.co.uk/news/liverpool-news/killing-mutilation-two-vulnerable-women-15291708 (accessed 1 September 2026).

[18] Benoit C, Jansson S M, Smith M, Flagg J. Prostitution stigma and its effect on the working conditions, personal lives, and health of sex workers. *J Sex Res* 2018; **55:** 457–471. 10.1080/00224499.2017.1393652 29148837

[19] Yang L, Wong L Y, Grivel M M, Hasin D S. Stigma and substance use disorders: an international phenomenon. *Curr Opin Psychiatry* 2017; **30:** 378–388. 10.1097/YCO.0000000000000351 PMC5854406 28700360

[20] Doughty J, Booth J, Smith M *et al*. Unmasking oral health stigma: a qualitative scoping review. *BMC Oral Health* 2025; DOI: 10.1186/s12903-025-07329-9. 10.1186/s12903-025-07329-9 PMC12922240 41408263

[21] Newell L-R, Fouillen K-J, Orliaguet M, Kichenin J, Boisramé S. Oral health effects of ecstasy (MDMA) and methamphetamine: a narrative review. *Front Oral Health* 2025; DOI: 10.3389/froh.2025.1645445. 10.3389/froh.2025.1645445 PMC12463819 41019449

[22] Shekarchizadeh H, Khami M R, Mohebbi S Z, Ekhtiari H, Virtanen J. Oral health of drug abusers: a review of health effects and care. *Iran J Public Health* 2013; **42:** 929–940. PMC4453891 26060654

[23] Teoh L, Moses G, McCullough M J. Oral manifestations of illicit drug use. *Aust Dent J* 2019; **64:** 213–222. 10.1111/adj.12709 31309583

[24] D'Amore M M, Cheng D, Kressin N R *et al*. Oral health of substance-dependent individuals: impact of specific substances. *J Subst Abuse Treat* 2011; **41:** 179–185. 10.1016/j.jsat.2011.02.005 PMC3384491 21474269

[25] Brondani M A, Alan R, Donnelly L. Stigma of addiction and mental illness in healthcare: the case of patients' experiences in dental settings. *PLoS One* 2017; **12:** e0177388. 10.1371/journal.pone.0177388 PMC5439661 28531176

[26] Coles E, Freeman R. Exploring the oral health experiences of homeless people: a deconstruction-reconstruction formulation. *Community Dent Oral Epidemiol* 2016; **44:** 53–63. 10.1111/cdoe.12190 26202302

[27] Folker L *et al*. Dental shame: a call for understanding and addressing the role of shame in oral health. *Community Dent Oral Epidemiol* 2026; **54:** 1–6. 10.1111/cdoe.70019 PMC12808861 40976871

[28] Benoit C, Ouellet N, Jansson M. Unmet health care needs among sex workers in five census metropolitan areas of Canada. *Can J Public Health* 2016; **107:** 266–271. 10.17269/CJPH.107.5178 PMC6972162 27763841

[29] British Dental Association. Dentists: 97% of New Patients Unable to Access NHS Care. 2024. Available at https://www.bda.org/media-centre/dentists-97-of-new-patients-unable-to-access-nhs-care/ (accessed 1 September 2026).

[30] Doughty J, Grossman A, Paisi M *et al*. A survey of dental services in England providing targeted care for people experiencing social exclusion: mapping and dimensions of access. *Br Dent J* 2022; DOI: 10.1038/s41415-022-4391-7. 10.1038/s41415-022-4391-7 PMC9208541 35725913

[31] Johnson L, Potter L C, Beeching H *et al*. Interventions to improve health and the determinants of health among sex workers in high-income countries: a systematic review. *Lancet Public Health* 2022; **8:** 141–154. 10.1016/S2468-2667(22)00252-3 PMC10564624 36334613

[32] Bradley N, Heidari E, Andreasson S, Newton T. Models of dental care for people experiencing homelessness in the UK: a scoping review of the literature. *Br Dent J* 2023; **234:** 816–824. 10.1038/s41415-023-5904-8 37291313

