# Supplementary material for: Survival, stigma and structural barriers: a qualitative study exploring the oral health experiences of women attending sex work support services in Liverpool
Source: Br Dent J. 2026 Oct 9;241(7):480–7. doi: 10.1038/s41415-026-0079-8 (PMC13652731; doi:10.1038/s41415-026-0079-8)
Supplement: Supplementary file 1 [file 41415_2026_79_MOESM2_ESM.docx]

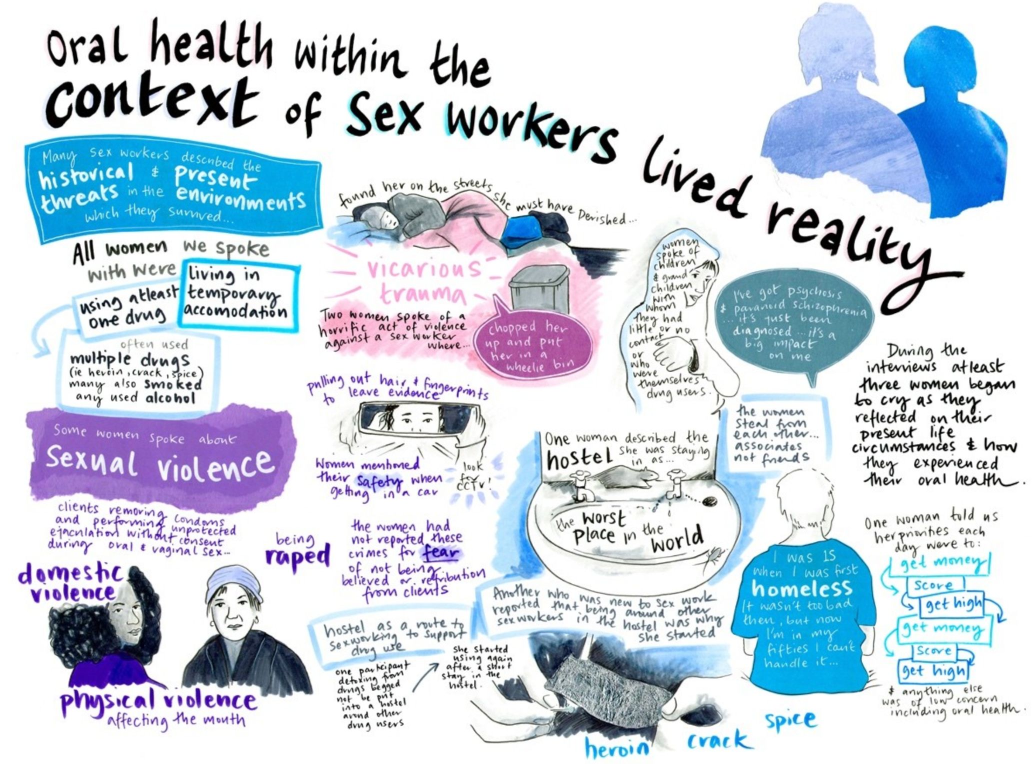
Oral health within the context of Sex workers lived reality
Many sex workers described the historical & present threats in the environments which they survived...
All women we spoke with were
using atleast one drug
living in temporary accomodation
often used multiple drugs (ie heroin, crack, spice) many also smoked any used alcohol
Some women spoke about Sexual violence
clients removing condoms and performing unprotected ejaculation without consent during oral & vaginal sex...
being raped
domestic violence
physical violence affecting the mouth
found her on the streets she must have perished...
vicarious trauma
Two women spoke of a horrific act of violence against a sex worker where...
chopped her up and put her in a wheelie bin
pulling out hair & fingerprints to leave evidence
Women mentioned their safety when getting in a car
look for CCTV!
the women had not reported these crimes for fear of not being believed or retribution from clients
hostel as a route to sexworking to support drug use
one participant detoxing from drugs begged not to be put into a hostel around other drug users
she started using again after a short stay in the hostel.
women spoke of children & grand children with whom they had little or no contact or who were themselves drug users.
I've got psychosis & paranoid schizophrenia ... it's just been diagnosed ... it's a big impact on me
the women steal from each other... associates not friends
One woman described the hostel she was staying in as...
the worst place in the world
Another who was new to sex work reported that being around other sex workers in the hostel was why she started
heroin
crack
spice
I was 15 when I was first homeless
It wasn't too bad then, but now I'm in my fifties I can't handle it...
During the interviews atleast three women began to cry as they reflected on their present life circumstances & how they experienced their oral health.
One woman told us her priorities each day were to:
get money
score
get high
get money
score
get high
& anything else was of low concern including oral health.

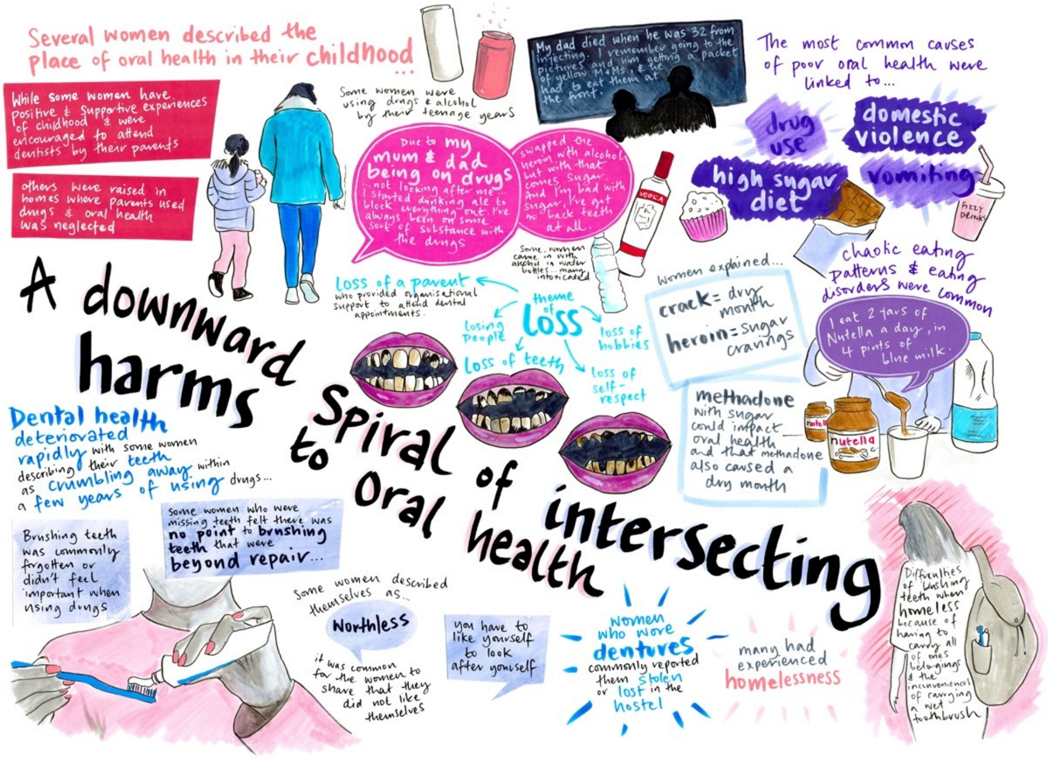
A downward Spiral of intersecting harms to Oral health
Several women described the place of oral health in their childhood...
While some women have positive & supportive experiences of childhood & were encouraged to attend dentists by their parents
others were raised in homes where parents used drugs & oral health was neglected
Some women were using drugs & alcohol by their teenage years
Due to my mum & dad being on drugs ...not looking after me I started drinking ale to block everything out. I've always been on some sort of substance with the drugs
swapped one heroin with alcohol but with that comes sugar. And I'm bad with sugar, I've got no back teeth at all.
Some women came in with alcohol in water bottles, many intoxicated
My dad died when he was 32 from injecting. I remember going to the pictures and him getting a packet of yellow M&Ms & we had to eat them at the front.
The most common causes of poor oral health were linked to...
drug use
domestic violence
high sugar diet
vomiting
fizzy drinks
chaotic eating patterns & eating disorders were common
loss of a parent who provided organisational support to attend dental appointments
theme of loss
losing people
loss of hobbies
loss of teeth
loss of self-respect
Women explained...
crack = dry mouth
heroin = sugar cravings
I eat 2 jars of Nutella a day, in 4 pints of blue milk.
methadone with sugar could impact oral health and that methadone also caused a dry mouth
nutella
Dental health deteriorated rapidly with some women describing their teeth as crumbling away within a few years of using drugs...
Brushing teeth was commonly forgotten or didn't feel important when using drugs
Some women who were missing teeth felt there was no point to brushing teeth that were beyond repair...
Some women described themselves as...
Worthless
it was common for the women to share that they did not like themselves
you have to like yourself to look after yourself
women who wore dentures commonly reported them stolen or lost in the hostel
many had experienced homelessness
Difficulties of brushing teeth when homeless because of having to carry all of one's belongings & the inconvenience of carrying a wet toothbrush

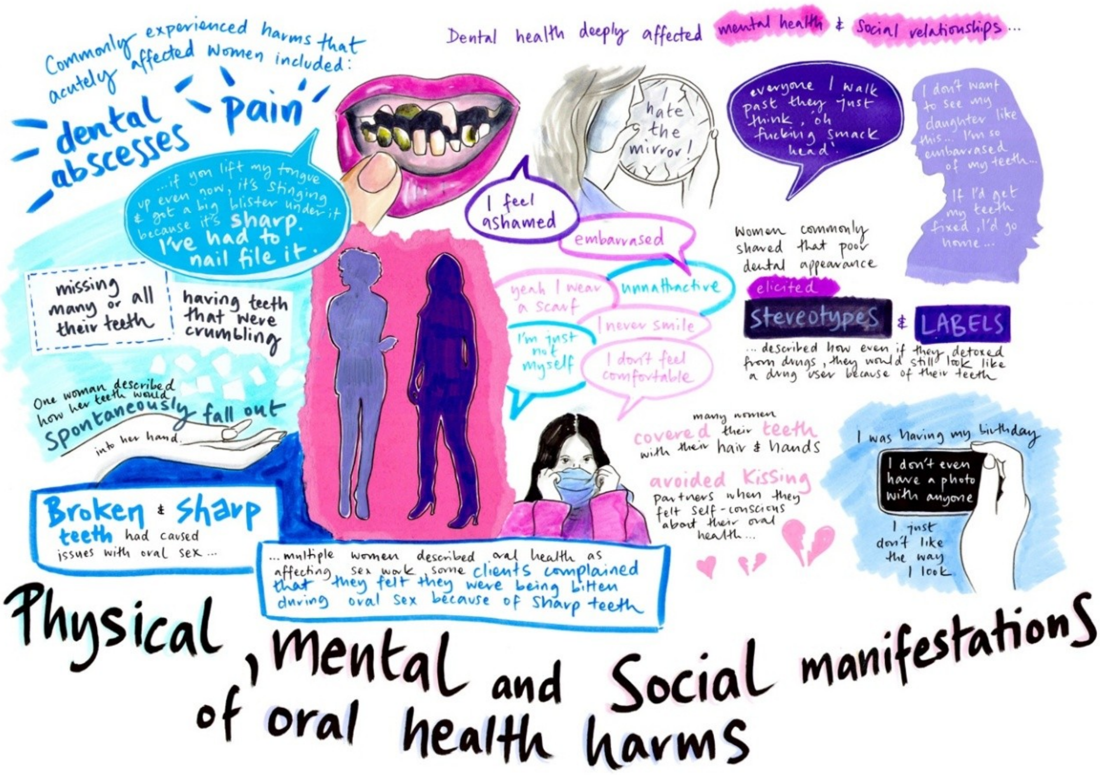

Commonly experienced harms that acutely affected women included:
dental abscesses
pain
...if you lift my tongue up even now, it's stinging & got a big blister under it because it's sharp. I've had to nail file it.
missing many or all their teeth
having teeth that were crumbling
One woman described how her teeth would spontaneously fall out into her hand
Broken & sharp teeth had caused issues with oral sex...
...multiple women described oral health as affecting sex work, some clients complained that they felt they were being bitten during oral sex because of sharp teeth
Dental health deeply affected mental health & social relationships...
I hate the mirror!
I feel ashamed
embarrassed
yeah I wear a scarf
unattractive
I never smile
I'm just not myself
I don't feel comfortable
everyone I walk past they just think, 'oh fucking smack head'.
I don't want to see my daughter like this... I'm so embarrassed of my teeth...
If I'd get my teeth fixed, I'd go home...
Women commonly shared that poor dental appearance elicited stereotypes & LABELS
...described how even if they detoxed from drugs, they would still look like a drug user because of their teeth
many women covered their teeth with their hair & hands
avoided kissing partners when they felt self-conscious about their oral health...
I was having my birthday
I don't even have a photo with anyone
I just don't like the way I look
Physical, mental and social manifestations of oral health harms

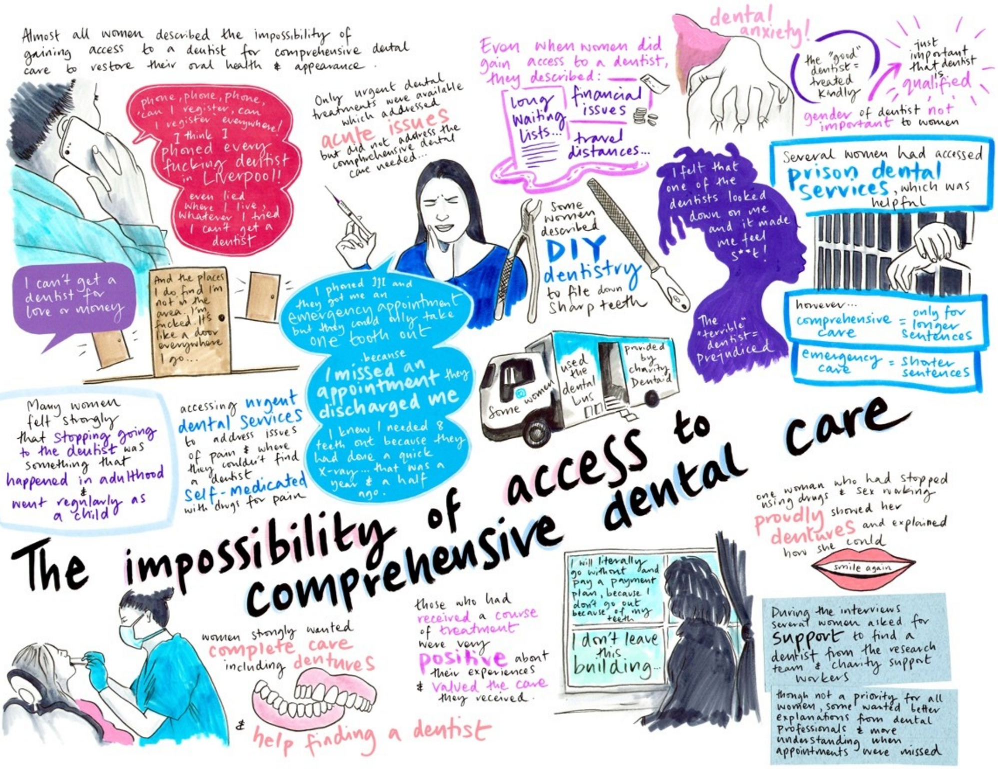

Almost all women described the impossibility of gaining access to a dentist for comprehensive dental care to restore their oral health & appearance.
phone, phone, phone, 'can I register, can I register' everywhere! I think I phoned every fucking dentist in Liverpool! even lied where I live, whatever I tried I can't get a dentist
I can't get a dentist for love or money
And the places I do find I'm not in the area. I'm fucked. It's like a door everywhere I go...
Only urgent dental treatments were available which addressed acute issues but did not address the comprehensive dental care needed...
I phoned 111 and they got me an emergency appointment but they could only take one tooth out because I missed an appointment they discharged me
I knew I needed 8 teeth out because they had done a quick X-ray... that was a year & a half ago.
Many women felt strongly that stopping going to the dentist was something that happened in adulthood & went regularly as a child
accessing urgent dental services to address issues of pain & where they couldn't find a dentist self-medicated with drugs for pain
Even when women did gain access to a dentist, they described:
long waiting lists...
financial issues
travel distances...
Some women described DIY dentistry to file down sharp teeth
Some women used the dental bus provided by charity Dentaid
dental anxiety!
the "good" dentist = treated kindly
just important that dentist is qualified
gender of dentist not important to women
I felt that one of the dentists looked down on me and it made me feel s**t!
The "terrible" dentist = prejudiced
Several women had accessed prison dental services, which was helpful
however... comprehensive care = only for longer sentences
emergency care = shorter sentences
The impossibility of access to comprehensive dental care
one woman who had stopped using drugs & sex working proudly showed her dentures and explained how she could smile again
women strongly wanted complete care including dentures & help finding a dentist
those who had received a course of treatment were very positive about their experiences & valued the care they received
I will literally go without and pay a payment plan, because I don't go out because of my teeth
I don't leave this building...
During the interviews several women asked for support to find a dentist from the research team & charity support workers
though not a priority for all women, some wanted better explanations from dental professionals & more understanding when appointments were missed

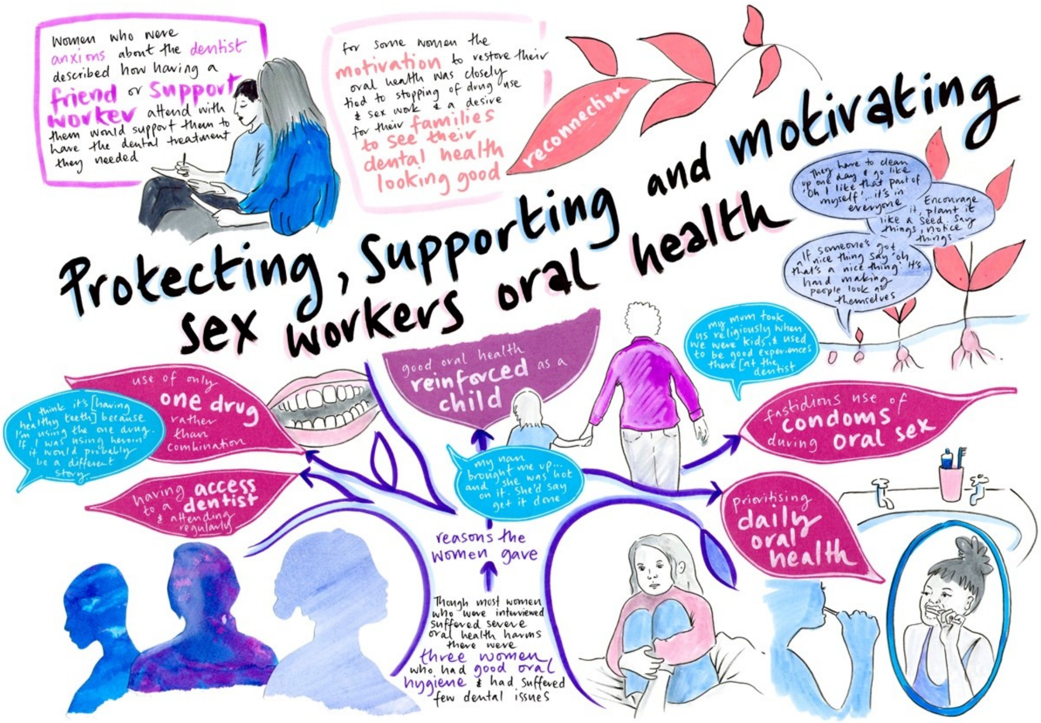

Protecting, Supporting and motivating sex workers oral health
Women who were anxious about the dentist described how having a friend or support worker attend with them would support them to have the dental treatment they needed
for some women the motivation to restore their oral health was closely tied to stopping of drug use & sex work & a desire for their families to see their dental health looking good
reconnection
They have to clean up one day & go like 'Oh I like that part of myself'... it's in everyone
Encourage it, plant it like a seed. Say things, notice things –
If someone's got a nice thing say 'oh that's a nice thing' It's hard making people look at themselves
my mum took us religiously when we were kids, & used to be good experiences there [at the dentist
use of only one drug rather than combination
I think it's [having healthy teeth] because I'm using the one drug. If I was using heroin it would probably be a different story.
good oral health reinforced as a child
fastidious use of condoms during oral sex
having access to a dentist & attending regularly
my nan brought me up... and she was hot on it. She'd say get it done
prioritising daily oral health
reasons the women gave
Though most women who were interviewed suffered severe oral health harms there were three women who had good oral hygiene & had suffered few dental issues
