# Supplementary material for: Survival, stigma and structural barriers: a qualitative study exploring the oral health experiences of women attending sex work support services in Liverpool
Source: Br Dent J. 2026 Oct 9;241(7):480–7. doi: 10.1038/s41415-026-0079-8 (PMC13652731; doi:10.1038/s41415-026-0079-8)
Supplement: Supplementary file 1 [file 41415_2026_79_MOESM1_ESM.docx]

**Oral health of women using sex worker support services study: Distress protocol**

Oral health can be an upsetting topic for people because it relates to appearance and also oral health can garner stigma which is associated with feelings of embarrassment or shame. Therefore, discussing oral health may present a sensitive topic for some participants. As a result, we have a distress protocol for the study which is as follows:

1 Indication of distress: If a participant indicates that they are experiencing a high level of stress / emotional distress or shows signs such as crying or facial expressions of distress then the interviewer will take action.

2. Interviewer actions: Stop Interview. Ask participant whether they are OK and offer emotional support. Assess whether further support is required from friends, family, social worker, GP or mental health charities.

3. Further actions: Discussion will continue only if participant confirms they are OK and wish to proceed. If they show high levels of distress interview will be stopped completely and emotional support continues as we as signposting to GP, social support network and contact again the next day to offer further support and check in.

4. Reporting of incident: Investigator (Janine Doughty) will document and report the incident to department lead.

We do not anticipate that dental professionals pose a risk of emotional distress during the interview process, but would follow the same process as above.
